# Complex ALK Fusions Are Associated With Better Prognosis in Advanced Non-Small Cell Lung Cancer

**DOI:** 10.3389/fonc.2020.596937

**Published:** 2020-12-11

**Authors:** Jin Kang, Xu-Chao Zhang, Hua-Jun Chen, Wen-Zhao Zhong, Yang Xu, Jian Su, Qing Zhou, Hai-Yan Tu, Zhen Wang, Chong-Rui Xu, Xue-Ning Yang, Zhi-Hong Chen, Xue Wu, Xian Zhang, Yang Shao, Yi-Long Wu, Jin-Ji Yang

**Affiliations:** ^1^ Division of Pulmonary Oncology, Guangdong Lung Cancer Institute, Guangdong Provincial People’s Hospital, Guangdong Academy of Medical Sciences, Guangzhou, China; ^2^ Medical Research Center, Guangdong Provincial People’s Hospital and Guangdong Academy of Medical Sciences, The Second School of Clinical Medicine, Southern Medical University, Guangzhou, China; ^3^ Guangdong Lung Cancer Institute, Guangdong Provincial Key Laboratory of Translational Medicine in Lung Cancer, Guangdong Provincial People’s Hospital & Guangdong Academy of Medical Sciences, School of Medicine, South China University of Technology, Guangzhou, China; ^4^ Translational Medicine Research Institute, Geneseeq Technology Inc., Toronto, ON, Canada; ^5^ Nanjing Geneseeq Technology Inc., Nanjing, China; ^6^ School of Public Health, Nanjing Medical University, Nanjing, China; ^7^ Medical Research Center, Guangdong General Hospital and Guangdong Academy of Medical Sciences, Guangzhou, China

**Keywords:** *EML4-ALK*, non-canonical *ALK* fusion, complex *ALK* fusions, tyrosine kinase inhibitors, non-small cell lung cancer

## Abstract

**Background:**

Echinoderm microtubule-associated protein-like 4 (*EML4*) is the canonical anaplastic lymphoma kinase (*ALK*) fusion partner in non-small cell lung cancer (NSCLC), and *ALK*-positive patients showed promising responses to ALK tyrosine kinase inhibitors (TKIs). However, studies that comprehensively investigate ALK TKI treatment in patients with different *ALK* fusion patterns are still lacking.

**Methods:**

Ninety-eight *ALK*-positive patients with advanced NSCLC were retrospectively studied for their response to crizotinib and subsequent treatments. Comprehensive genomic profiling (CGP) was conducted to divide patients into different groups based on their *ALK* fusion patterns. Non-canonical *ALK* fusions were validated using RNA-sequencing.

**Results:**

54.1% of patients had pure canonical *EML4-ALK* fusions, 19.4% carried only non-canonical *ALK* fusions, and 26.5% harbored complex *ALK* fusions with coexisting canonical and non-canonical *ALK* fusions. The objective response rate and median progression-free survival to crizotinib treatment tended to be better in the complex *ALK* fusion group. Notably, patients with complex *ALK* fusions had significantly improved overall survival after crizotinib treatment (p = 0.012), especially when compared with the pure canonical *EML4-ALK* fusion group (p = 0.010). The complex *ALK* fusion group also tended to respond better to next-generation ALK TKIs, which were used as later-line therapies. Most identified non-canonical *ALK* fusions were likely to be expressed in tumors, and some of them formed canonical *EML4-ALK* transcripts during mRNA maturation.

**Conclusion:**

Our results suggest NSCLC patients with complex *ALK* fusions could potentially have better treatment outcomes to ALK TKIs therapy. Also, diagnosis using CGP is of great value to identify novel *ALK* fusions and predict prognosis.

## Introduction

Lung cancer is the leading cause of cancer-related mortality worldwide and non-small cell lung cancer (NSCLC) accounts for more than 80% of all diagnosed cases (1, 2). Approximately 2–7% NSCLC patients harbor anaplastic lymphoma kinase (*ALK*) gene rearrangements (3, 4), leading to aberrant expression and oncogenic activation of *ALK*. Echinoderm microtubule-associated protein-like 4 (*EML4*)-*ALK* is the canonical and most common *ALK* gene arrangement found in NSCLC, by which multiple *EML4* breakpoints fuse in frame with the kinase domain of *ALK* (5). Indeed, more than 15 different *EML4*-*ALK* fusion variants have been reported in NSCLC, with v1, v2, and v3a/b being the most abundant variants (6). Some *ALK* fusions that were less commonly reported in NSCLC (i.e., non-canonical *ALK* fusions) include kinesin family member 5B (*KIF5B*)-*ALK*, TRK-fused gene (*TFG*)-*ALK*, kinesin light chain 1 (*KLC1*)-*ALK*, striatin (*STRN*)-*ALK*, and TNFAIP3 interacting protein 2 (*TNIP2*)-*ALK* (7–10), while some *ALK* fusions were mainly found in other cancers, for example, nucleophosmin (*NPM*)-*ALK* fusion was almost exclusively found in large cell lymphomas (11).

Due to the rapid progress in targeted therapy, tyrosine kinase inhibitors (TKIs) are becoming the standard of care for oncogene*-*positive NSCLC. Crizotinib, showed improved objective response rate (ORR), progression-free survival (PFS), and overall survival (OS) in *ALK*-positive NSCLC patients compared with chemotherapy (12–14). Subsequent generations of ALK TKIs were then developed and showed promising clinical responses (15–17). Nevertheless, about 10–40% of *ALK*-positive NSCLC patients failed to respond to ALK TKIs, suggesting that further stratifying *ALK*-positive patients based on their TKI response is of clinical importance. Given that *EML4*-*ALK* is the most common *ALK* fusions in NSCLC, several studies demonstrated that different variants of *EML4*-*ALK* fusions have distinct sensitivity to ALK inhibitors (18, 19), although some researchers found there was no significant differences in PFS among patients with these *EML4*-*ALK* variants (20). In contrast, there are limited data about the TKI clinical response for canonical (*EML4-ALK*) versus non-canonical (non-*EML4-ALK*) fusions in NSCLC. Rosenbaum *et al*. compared 14 canonical *ALK* fusions with 3 non-canonical *ALK* fusions and concluded that patients with canonical *ALK* fusions had better overall survival (OS) (21). However, this study is limited by small patient numbers and needs to be validated in larger patient cohorts.

Unlike traditional diagnosis methods, such as break-apart fluorescence *in situ* hybridization (FISH) and immunohistochemistry (IHC), which only give the positivity/negativity of *ALK* fusion, comprehensive genomic profiling (CGP) is able to separate different *ALK* fusion variants and identify rare fusion partners that may be associated with different sensitivities to ALK TKIs. In the current study, we used CGP to characterize 98 *ALK*-positive NSCLC patients and grouped them based on the presence of canonical and/or non-canonical *ALK* fusions. We aimed to study the crizotinib response in patients with different *ALK* fusion patterns and sought to correlate the clinical outcomes with different patient/treatment characteristics and genomic profiling results.

## Materials and Methods

### Patients and Methods

This study was approved by the institutional ethics review board of Guangdong Provincial People’s Hospital [Ethics number: No. GDREC2019323H (R1)]. All patients signed informed consent forms prior to sample collection and consented for publication of related clinical information and any accompanying image. Ninety-eight *ALK*-positive patients with advanced NSCLC were retrospectively studied. Hybridization capture-based CGP using next-generation sequencing (NGS) was performed with (FFPE) or plasma samples collected at baseline (n = 43) or progressive disease (PD; n = 55) to characterize their *ALK* fusion patterns. Crizotinib clinical response was evaluated *via* computed tomography scans six weeks after the first crizotinib administration and every 6/8 weeks thereafter according to Response Evaluation Criteria in Solid Tumors (RECIST) version 1.1. PFS was measured from the date of initiation of crizotinib treatment until disease progression or death. Overall survival (OS) was calculated from the date of initiation of crizotinib treatment to death resulting from any causes or was censored at the last follow-up on November 30, 2019.

### DNA Extraction, Library Preparation, and CGP Data Analysis

Tumor genomic DNA was extracted from FFPE samples with a tumor content >50% using a QIAamp DNA FFPE Kit (Qiagen, Hilden, Germany) to detect somatic mutations. Genomic DNA from white blood cells was extracted using DNeasy Blood & Tissue kit (Qiagen, Hilden, Germany). Hybridization capture-based CGP using NGS was performed at two genetic testing centers. Brieﬂy, the KAPA Hyper Prep Kit (Kapa Biosystems, USA) was used for DNA library preparation. Customized xGen lockdown probes (Integrated DNA Technologies, USA) were used for hybridization enrichment. All procedures were conducted according to the manufacturers’ instructions. The overlapping 279 cancer-relevant genes from the two testing centers were included for CGP analysis (**Supplementary Table 1**). Somatic mutations were first filtered for common single nucleotide polymorphisms (SNPs) with dbSNP and 1,000 Genome datasets, followed by further filtration of germline mutations with normal blood controls. Structural variants were detected using FACTERA with default parameters (22). The fusion reads were further manually reviewed and conﬁrmed on Integrative Genomics Viewer (IGV) (23). ADTEx (http://adtex.sourceforge.net) was used to identify copy number variations (CNVs) with default parameters.

### Break-Apart Fluorescence *In Situ* Hybridization (FISH) and Immunohistochemistry (IHC)

Unstained FFPE sections from tumor specimens collected at diagnosis were subjected to FISH with *ALK* break-apart probes (Vysis ALK Break Apart FISH Probe Kit; Abbott Molecular, Abbot Park, IL, USA) and/or IHC staining with Ventana anti-ALK (D5F3) rabbit monoclonal primary antibody (Roche Diagnostics, Mannheim, Germany), following the manufacturers’ instructions.

### Reverse Transcriptase-Polymerase Chain Reaction (RT-PCR) and Sanger Sequencing

Total RNA from FFPE samples was extracted using RNeasy FFPE kit (QIAGEN). Reverse transcription was performed with Superscript Vilo mastermix (Life Technologies). Gel-puriﬁed DNA was sent for Sanger sequencing to identify the sequence in cDNA.

### RNA-Sequencing (RNA-Seq)

Poly(A) fractions from the globin depleted RNA samples (1.0 μg) were purified by oligo-dT purification beads (Illumina, Inc., San Diego, USA) and then used to construct cDNA libraries following the TruSeq RNA Sample Preparation Guide (Illumina, Inc., San Diego, USA). Sequencing was performed on the HiSeq 2000 System (Illumina, Inc.) using the TruSeq Paired-End (PE) 100 bp Kit (Illumina, Inc.). Real-time analysis and base calling were conducted using the Control software in the instrument. The initial processing of reads from the HiSeq instrument used the Illumina CASAVA (v1.8).

### Statistical Analysis

The comparison of mutation frequency between different *ALK* fusion groups was done using Fisher’s exact test, and genes with p values smaller than 0.1 were included for further analysis. For survival data, Kaplan-Meier curves were analyzed using the log-rank test; for the pairwise log-rank test, the p values were adjusted by Benjamini and Hochberg method; the censored points were marked in the figure when the patient loss to follow-up during the study. The univariate and multivariate analyses were performed using the Cox regression model. For analyzing the next generation TKIs, only patients who had known next generation TKI treatment history were included. Two-sided p values of less than 0.05 were considered as statistically significant. All statistical analyses were done in R (v.3.6.0).

## Results

### Patient Clinical Characteristics and *ALK* Fusion Patterns

From January 2016 to June 2019, a total of 2016 NSCLC patients from our hospital were diagnosed with NSCLC, and 150 of them (7.4%) were detected to be *ALK*-positive using break-apart FISH and/or IHC. Ninety-eight *ALK*-positive patients with advanced NSCLC were retrospectively studied for their clinical response to crizotinib after excluding patients with early staging, unacceptable crozotinib toxicitiesor unclear clinical history, as well as patients without crizotinib treatment (**Supplementary Figure 1**). The *ALK* fusion patterns were characterized using CGP, with 43 patients being sequenced at diagnosis (baseline) and 55 patients being sequenced at PD after crizotinib treatment (**Supplementary Figure 1**). Since the time of sampling (i.e., baseline vs. PD) makes little difference on the frequency of various *ALK* fusion patterns (**Supplementary Figure 2A** vs. **2B**), we combined all the CGP analysis and used it to divide all 98 *ALK*-positive patients into 3 groups (**Supplementary Figure 2B**): 1) 53 patients (54.1%) had only the canonical *EML4-ALK* fusions; 2) 19 patients (19.4%) carried only the non-canonical *ALK* fusions; 3) 26 patients (26.5%) who harbored both canonical and non-canonical *ALK* fusions were classified as the complex *ALK* fusion group. As shown in **Table 1** and **Supplementary Table 2**, patient characteristics such as age, gender, smoking history, histology, performance status (PS) scores, and disease stage were similar across different *ALK* fusion groups, with majorities of the *ALK*-positive patients in our cohort were never smokers (81.6%) with lung adenocarcinoma (ADC; 96.0%). Also, most patients received crizotinib as the first line (63.3%) or second-line (28.6%) treatment. After disease progression to crizotinib treatment, more than 60% of the patients used next-generation ALK inhibitors and about 40% of patients received palliative treatment (**Table 1**). The median OS for all 98 patients was 19.7 months.

**Table 1:** Demographics and clinicopathologic characteristics of *ALK*-positive NSCLC patients.

	Pure *EML4-ALK* fusionsn (%)	Pure uncommon*ALK* fusionsn (%)	Complex*ALK* fusionsn (%)	All patientsn (%)
**Number of patients**	53	19	26	98
**Median age, years (range)**	46 (25–76)	50 (30–69)	49 (28–70)	47.5 (25–76)
**Gender**				
Male	22 (41.5)	10 (52.6)	15 (57.7)	47 (48.0)
Female	31 (58.5)	9 (47.4)	11 (42.3)	51 (52.0)
**Smoking history**				
Yes	10 (18.9)	4 (21.1)	4 (15.4)	18 (18.4)
No	43 (81.1)	15 (78.9)	22 (84.6)	80 (81.6)
**Histology**				
ADC	51 (96.2)	18 (94.7)	25 (96.2)	94 (96.0)
SCC	0 (0)	0 (0)	1 (3.8)	1 (1.0)
LCNEC	1 (1.9)	0 (0)	0 (0)	1 (1.0)
ASC	1 (1.9)	1 (5.3)	0 (0)	2 (2.0)
**Disease stage**				
III	3 (5.7)	1 (5.3)	1 (3.8)	5 (5.1)
IV	50 (94.3)	18 (94.7)	25 (96.2)	93 (94.9)
**PS score**				
0	2 (3.8)	0 (0)	0 (0)	2 (2.0)
1	46 (86.8)	17 (89.5)	23 (88.5)	86 (87.8)
2	5 (9.4)	2 (10.5)	3 (11.5)	10 (10.2)
**Crizotinib (line of treatment)**				
1	34 (64.2)	11 (57.9)	17 (65.4)	62 (63.3)
2	14 (26.4)	7 (36.8)	7 (26.9)	28 (28.6)
3	5 (9.4)	0 (0)	2 (7.7)	7 (7.1)
5	0 (0)	1 (5.3)	0 (0)	1 (1.0)
**Best clinical response for criztinib**				
CR	0 (0)	1 (5.3)	0 (0)	1 (1.0)
PR	35 (66.0)	12 (63.2)	20 (76.9)	67 (68.4)
SD	13 (24.5)	4 (21.1)	3 (11.5)	20 (20.4)
PD	5 (9.4)	2 (10.5)	3 (11.5)	10 (10.2)
**Post-crizotinib ALK inhibitor treatment**				
Yes	36 (67.9)	12 (63.2)	15 (57.7)	63 (64.3)
No	11 (20.8)	2 (10.5)	3 (11.5)	16 (16.3)
NA	6 (11.3)	5 (26.3)	8 (30.8)	19 (19.4)
Palliative treatment for advanced NSCLC*				
Yes	21 (39.6)	12 (63.2)	8 (30.8)	41 (41.8)
No	31 (58.5)	7 (36.8)	16 (61.5)	54 (55.1)
NA	1 (1.9)	0 (0)	2 (7.7)	3 (3.1)
**Baseline brain metastasis**				
Yes	10 (18.9)	5 (26.3)	8 (30.8)	23 (23.5)
No	43 (81.1)	14 (73.7)	18 (69.2)	75 (76.5)

*Palliative treatments include local surgical therapy, palliative radiotherapy, and interventional therapy.

### The Association Between *ALK* Fusion Status and Crizotinib Treatment Outcomes

Firstly, we assessed the drug response in 43 *ALK*-positive patients with baseline CGP. As shown in **Supplementary Table 3**, the ORR for crizotinib was 65.1% and the disease control rate (DCR) was 83.7%. By examining the crizotinib response in each *ALK* fusion group, we found that DCR was similar among all groups while the complex *ALK* fusion group had improved ORR compared with other groups (**Supplementary Table 3**). Similar results were obtained when we used all 98 patients whose *ALK* fusion pattern was determined by combining baseline and progressive disease CGP (**Table 1** and **Supplementary Table 3**).

**Figure 1:**
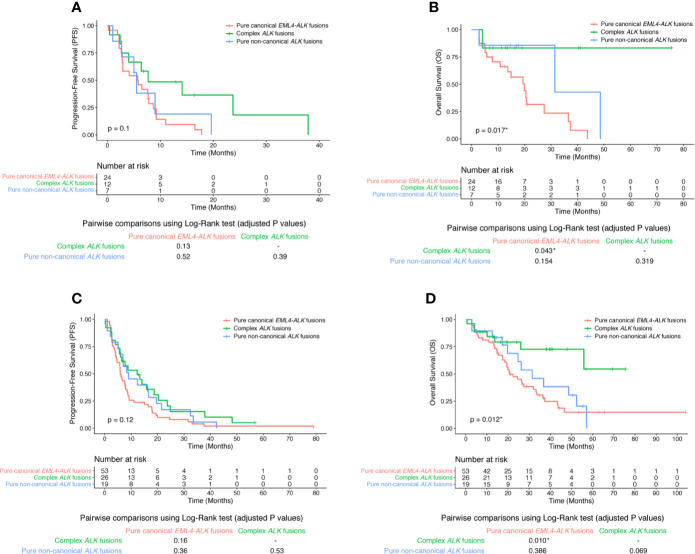
The clinical response of crizotinib in different *ALK* fusion groups. Kaplan-Meier curve of PFS **(A)** or OS **(B)** of crizotinib treatment in 43 patients with baseline CGP in strata of different *ALK* fusion patterns. Kaplan-Meier curve of PFS **(C)** or OS **(D)** of crizotinib treatment in all 98 *ALK*-positive patients in strata of different *ALK* fusion patterns. Log-rank test was used to analyze the OS or PFS for all 3 groups (The p value was shown within the Kaplan-Meier curve). Benjamini and Hochberg (BH)-adjusted p values of the log-rank test were reported for all pairwise comparisons (Individual pairwise comparison p values were shown below the Kaplan-Meier curve).

We further examined the post-treatment patient survival in these *ALK*-positive patients. For the 43 patients with baseline CGP, there was no statistically significant difference in PFS among patients with different *ALK* fusion patterns (log-rank p value = 0.1; **Figure 1A**). Intriguingly, complex *ALK* fusions were significantly associated with better overall survival (OS) than other *ALK* fusion patterns (log-rank p value = 0.017), especially when comparing the complex *ALK* fusion group with the pure canonical *EML-ALK* fusion group (pairwise log-rank p values were 0.043; **Figure 1B**). The results became even more significant if we included all 98 patients. Despite the statistically indistinguishable PFS among 3 *ALK* fusion groups (log-rank p value = 0.12; **Figure 1C**), patients with complex *ALK* fusions were likely to have better OS than patients with pure canonical *EML-ALK* fusions (pairwise log-rank p value = 0.01; **Figure 1D**). Therefore, our data suggest that harboring complex *ALK* fusions was a potential positive biomarker for crizotinib treatment in advanced NSCLC patients. Also, because analysis based on baseline CGP (n = 43) and analysis based on the combination of baseline and post-crizotinib CGP (n = 98) gave similar results in terms of the frequency of various *ALK* fusion patterns and the clinical results, we used the data of all 98 *ALK*-positive patients for the later on analysis.

### The Correlation Between the Crizotinib Response and the Clinical/Mutational Characteristics

**Figure 2:**
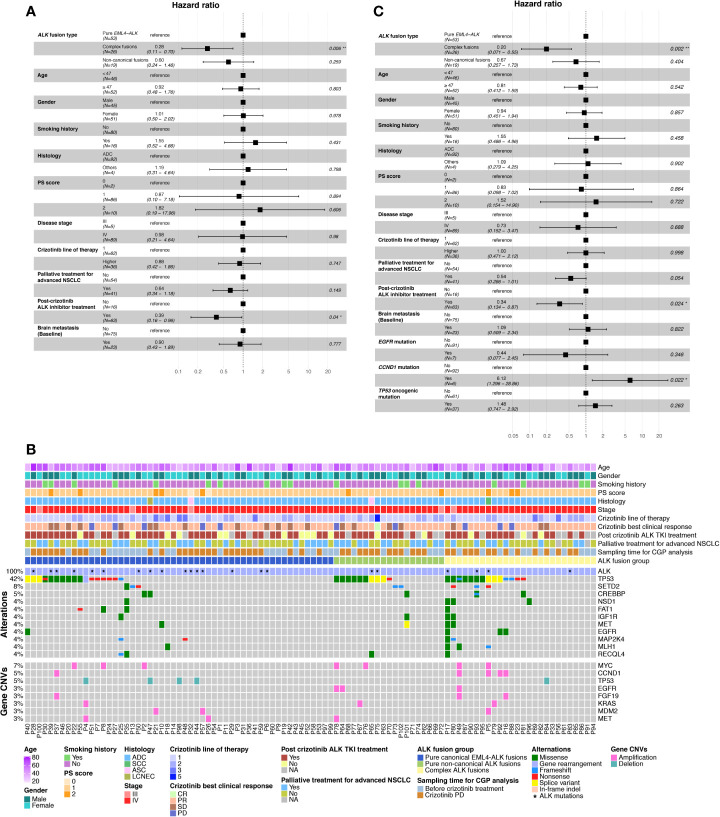
The correlation between the crizotinib response and the clinical/mutational characteristics. **(A)** Forest plot of multivariate Cox regression analysis demonstrating the association between different clinical characteristics and OS in 98 *ALK*-positive NSCLC patients after crizotinib treatment. **(B)** Top changed genomic features in 98 NSCLC patients. Patient clinicopathologic characteristics (upper panel), co-mutation plot of genetic alterations (middle panel), and gene-level copy-number variation (lower panel) were illustrated. Genes were ranked based on the number of alterations. CNV, copy-number variation; PS score, performance status score; CR, complete response; PR, partial response; SD, stable disease; PD, progressive disease. **(C)** Forest plot of multivariate Cox regression analysis demonstrating the association between clinical/mutational characteristics and OS in 98 *ALK*-positive NSCLC patients after crizotinib treatment.

Next, we investigated the correlation between patients’ post-crizotinib OS and other demographic/clinicopathologic characteristics. As illustrated in **Supplementary Table 4**, complex *ALK* fusions and post-crizotinib ALK inhibitor treatment were the only 2 factors that were significantly associated with improved OS (univariate Cox regression analysis, p values were 0.005 and 0.018, respectively). By multivariate analysis, we found complex *ALK* fusions and post-crizotinib ALK inhibitor treatment still significantly correlated with OS (**Figure 2A**). These results imply that harboring complex *ALK* fusions or subsequently treating with next-generation ALK TKIs are likely to associate with prolonged post-crizotinib survival in these *ALK*-positive patients.

We then checked the somatic mutation profile associated with different *ALK* fusion patterns. Tumor protein p53 (*TP53*) mutation/deletion and *MYC* amplification were found to be the most frequent genomic alterations in each *ALK* fusion group, followed by genomic changes in SET domain containing 2 (*SETD2*), CREB binding protein (*CREBBP*), epidermal growth factor receptor (*EGFR*), and cyclin D1 (*CCND1*) (**Figure 2B**). When comparing mutation frequency between different *ALK* fusion groups, *EGFR* mutation/amplification (Fisher’s exact test, p value = 0.038) and *CCND1* amplification (Fisher’s exact test, p value = 0.087) were the top 2 genomic alterations enriched in complex *ALK* fusion groups compared with the pure canonical *EML4-ALK* fusion group (**Supplementary Table 5**). To rule out the possibility that the improved OS in the complex *ALK* fusion group was due to the treatment effects from other targeted drugs (e.g., treating *EGFR* mutation/amplification-positive patients with EGFR TKIs), we included the mutation/CNV status of *EGFR* and *CCND1* into the multivariate Cox regression analysis. We also included the oncogenic/loss-of-function *TP53* mutations given that they have been shown to be associated with unfavorable treatment outcomes in *ALK*-positive NSCLC. Complex *ALK* fusions and post-crizotinib ALK inhibitor treatment could still predict post-crizotinib OS after including these genomic alterations (p values were 0.002 and 0.024, respectively); *EGFR* mutation/amplification was not significantly associated with OS, whereas *CCND1* amplification was likely to be a hazard factor for OS (**Figure 2C**).

Lastly, we checked whether some acquired molecular features may explain the differential overall survival between the complex *ALK* fusion group and the other groups. Among 98 *ALK*-positive patients in our cohort, 17 of them had both baseline and crizotinib-PD CGP analysis, including 10 patients with pure canonical *EML4-ALK* fusions, 4 with pure non-canonical *ALK* fusions, and 3 patients with complex *ALK* fusions. Interestingly, nearly all the acquired *ALK* resistant mutations to ALK TKIs were found in the pure canonical *EML4-ALK* fusion group, implying the potential association between TKI resistant mechanisms and *ALK* fusion patterns (**Supplementary Figure 3**).

### Complex *ALK* Fusions Also Had a Better Post-Crizotinib OS After the Next-Generation ALK TKIs Treatment

**Figure 3:**
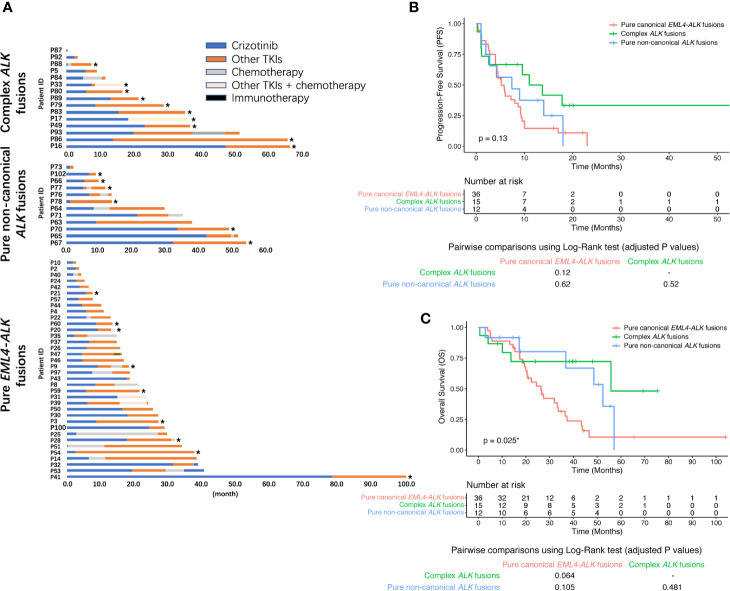
Therapeutic response to next-generation ALK TKIs in post-crizotinib patients. **(A)** Swimmer plot demonstrating the post-crizotinib treatment history in 63 NSCLC patients. The asterisk represents ongoing treatment with the last follow-up on November 30, 2019. Kaplan-Meier curve of PFS **(B)** or OS **(C)** in 63 next-generation ALK TKI-treated NSCLC patients in strata of different *ALK* fusions. When multiple next-generation ALK TKIs were used after crizotinib, the ALK TKI that immediately followed crizotinib treatment was included for the analysis. The OS was calculated from the date of initiation of crizotinib treatment to death resulting from any causes or was censored at the last follow-up. BH-adjusted p values of the log-rank test were reported for pairwise comparisons.

Given both complex *ALK* fusions and post-crizotinib ALK inhibitor treatment could predict post-crizotinib OS, we then studied whether patients with complex *ALK* fusions were more likely to respond to next-generation ALK TKIs. Of 98 *ALK*-positive patients, more than half of them were known to receive second- and/or third-generation ALK TKIs (**Figure 3A**). For patients with pure canonical *EML4-ALK* fusions, 6 patients switched to alectinib (median PFS = 5.0 months), 5 patients took brigatinib (median PFS = 5.2 months), 9 patients received ceritinib (median PFS = 5.8 months), and 7 patients received foritinib (median PFS = 5.2 months). The remaining 3 pure canonical *EML4-ALK* fusion patients received ensartinib (PFS = 4.5 months), lorlatinib (PFS = 5.0 months), and foritinib plus chemotherapy (PFS = 8.7 months), respectively. In the pure non-canonical *ALK* fusion group, 3 patients switched to alectinib (median PFS = 9.0), 2 patients took brigatinib (median PFS = 1.1 months), 1 patient received foritinib (median PFS = 11.6 months), 1 patient received ceritinib (PFS = 14.0 months), and 1 patient was treated with apatinib (PFS = 1.2 months). Among the complex *ALK* fusions cohort, 2 patients switched to brigatinib (PFS = 52.8 months and 1.0 month, respectively), 2 patients took ceritinib (PFS = 11.0 months and 17.8 months, respectively), 3 patients received aletinib (the duration of 2 patients was less than 1 month, and 1 patient have not progressed until the last follow-up), 5 patients treated with foritinib (clinical trial NCT04237805; median PFS = 13.7 months), and 1 patient received foritinib and concurrent chemotherapy (PFS > 18.2 months). As shown in **Figures 3A, B**, the complex *ALK* fusion group tended to have better response to next-generation ALK TKIs than other groups, although the PFS was not statistically significant (log-rank p value = 0.13). Similarly, these patients also seemed to have a better OS (log-rank p value = 0.025; **Figure 3C**). These results imply that patients with complex *ALK* fusions might have a better chance to respond to next-generation ALK TKIs after crizotinib treatment, which might partially contribute to their improved OS.

### Validation of Non-Canonical *ALK* Fusions

Lastly, we investigated the non-canonical *ALK* fusions to check if they could form functional *ALK* fusion products. By CGP analysis, we identified multiple novel non-canonical *ALK* fusion partners, including dystrophin (*DMD*), transmembrane protein 178A (*TMEM178A*), spectrin repeat containing nuclear envelope protein 1 (*SYNE1*), zinc finger CCCH-type containing 8 (*ZC3H8*), acireductone dioxygenase 1 (*ADI1*), AF4/FMR2 family member 3 (*AFF3*), protein kinase C epsilon (*PRKCE*), CUGBP Elav-like family member 4 (*CELF4*), mal T-cell differentiation protein-like (*MALL*), SET binding factor 2 (*SBF2*), proteasome 20S subunit alpha 8 (*PSMA8*), potassium voltage-gated channel modifier subfamily G member 3 (*KCNG3*), peroxidasin (*PXDN*), and ring finger protein 10 (*RNF10*) (**Table 2**). Most samples with the pure non-canonical *ALK* fusions had positive IHC, indicating that most of the identified non-canonical *ALK* fusions were likely to express the fusion products in the tumor (**Table 2**).

**Table 2:** The list of known or novel non-canonical *ALK* gene fusions identified in the NSCLC patient cohort.

	*ALK* Fusion Partner	*ALK* Fusion Group	Fusion Site	FISH	IHC (Ventana)
**Known *ALK* fusions**	*GALNT14*	Pure non-canonical *ALK* fusions	*GALNT14-ALK* (exon1:exon19)	+	+
	*HIP1*	Pure non-canonical *ALK* fusions	*HIP1-ALK* (exon19:exon20)	+	+
	*HIP1*	Complex *ALK* fusions	*HIP1-ALK* (exon1:exon16)	NA*	+
	*HIP1*	Pure non-canonical *ALK* fusions	*HIP1-ALK* (exon19:exon19)	NA	+
	*KIF5B*	Pure non-canonical *ALK* fusions	*KIF5B-ALK* (exon17:exon20)	+	NA
	*SETD2*	Pure non-canonical *ALK* fusions	*SETD2-ALK* (exon1:exon20)	NA	+
	*KLC1*	Pure non-canonical *ALK* fusions	*KLC1-ALK* (exon9:exon20)	+	+
	*BIRC6*	Complex *ALK* fusions	*BIRC6-ALK* (exon43:exon19)	+	+
	*LOC728730*	Complex *ALK* fusions	*LOC728730-ALK* (exon5:exon20)	NA	+
	*CRIM1*	Complex *ALK* fusions	*CRIM1-ALK* (exon2:exon20)	+	+
	*CLIP4*	Complex *ALK* fusions	*CLIP4-ALK* (exon1:exon20)	+	NA
	*PPP1R21*	Complex *ALK* fusions	*PPP1R21-ALK* (exon8:exon20)	+	+
**Novel *ALK* fusions**	*DMD*	Pure non-canonical *ALK* fusions	*DMD-ALK* (exon55:exon20)	+	+
	*ALK*	Pure non-canonical *ALK* fusions	*ALK-ALK* (intron1:intron19)	+	+
	*TMEM178A*	Pure non-canonical *ALK* fusions	*TMEM178A-ALK* (exon1:exon20)	NA	+
	*SYNE1*	Pure non-canonical *ALK* fusions	*SYNE1-ALK* (exon63:exon20)	NA	+
	*ZC3H8*	Pure non-canonical *ALK* fusions	*ZC3H8-ALK* (exon8:exon20)	+	NA
	*ADI1*	Pure non-canonical *ALK* fusions	*ADI1-ALK* (exon2:exon20)	+	NA
	*AFF3*	Pure non-canonical *ALK* fusions	*AFF3-ALK* (exon12:exon20)	NA	+
	*PRKCE*	Complex *ALK* fusions	*PRKCE-ALK* (exon10:exon20)	NA	+
	*CELF4*	Complex *ALK* fusions	*CELF4-ALK* (exon2:exon20)	+	NA
	*MALL*	Complex *ALK* fusions	*MALL-ALK* (exon1:exon20)	+	+
	*SBF2*	Complex *ALK* fusions	*SBF2-ALK* (exon1:exon18)	+	NA
	*PSMA8*	Complex *ALK* fusions	*PSMA8-ALK* (exon2:exon18)	+	+
	*KCNG3*	Complex *ALK* fusions	*KCNG3-ALK* (exon1:exon20)	+	NA
	*PXDN*	Complex *ALK* fusions	*PXDN-ALK* (exon1:exon20)	+	NA
	*RNF10*	Pure non-canonical *ALK* fusions	*RNF10-ALK* (exon1:exon19)	NA	+

*NA, Results not available due to lack of testing information.

**Figure 4:**
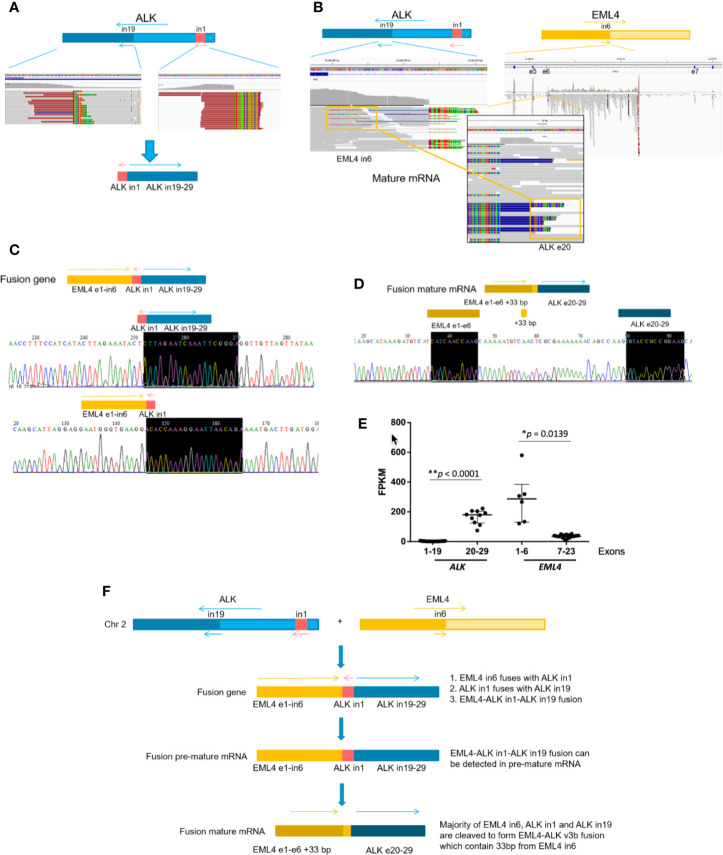
Non-canonical *ALK* fusions detected by CGP in an NSCLC patient (P64) resulted in a canonical *EML4-ALK* fusion mRNA. **(A)** *ALK* intron1-*ALK* intron19 fusion was detected at DNA levels by DNA-sequencing (DNA-Seq). **(B)** Mature *EML4-ALK* v3b fusion was detected at RNA levels by mRNA-seq. **(C)** Validation of *EML4-ALK* intron1 fusion and *ALK* intron1-*ALK* intron19 fusion at DNA levels, respectively, by PCR amplification of the fusion region followed by Sanger Sequencing. **(D)** RT-PCR validation of *EML4-ALK* v3b fusion at mRNA levels. **(E)** The relative expression level of *ALK* and *EML4* detected by RNA-seq. FPKM: Fragments per kilobase of transcript per Million mapped reads. **(F)** Model for stepwise *EML4-ALK* fusion formation during gene transcription.

We then selected several novel *ALK* fusions for further studies. Patient P64 had a rare *ALK* fusion, linking *ALK* intron1 with *ALK* intron19 (**Figure 4A**). We detected mature *EML4-ALK* (v3b) mRNA using RNA-seq (**Figure 4B**). The CGP and RNA-seq results were further validated using PCR and RT-PCR, respectively (**Figures 4C, D** **)**, and mRNA expression level of *EML4* exon1-6 and *ALK* exon20-29, which corresponds to v3b variant of *EML4-ALK* fusion, was also significantly higher than other exons of these two genes (**Figure 4E**). To rule out the possibility that *ALK* intron1-intron19 fusion and *EML4-ALK* fusion independently existed in the patient sample while CGP failed to detect the latter, we searched through the DNA sequencing and RNA-seq data and found the evidence of fusing *EML4* intron6*-ALK* intron1-*ALK*-intron19 at both DNA and pre-mature mRNA levels (**Figure 4C** and **Supplementary Figure 4**). These results indicate that *EML4* intron6*-ALK* intron1-*ALK*-intron19 was fused together in patient P64, and *ALK* intron1 was spliced out during mRNA maturation, resulting in the canonical *EML4-ALK* fusion (**Figure 4F**). Moreover, Patient P62 carried *GALNT14*-*ALK* fusion and *SLC19A3* intergenic region (IGR)-*ALK* fusion simultaneously (**Supplementary Figures 5A, B** **)**. We detected both *EML4* intron13*-GALNT14* fusion and *GALNT14*-*ALK* exon19 fusion in pre-mature mRNA by RNA-seq (**Supplementary Figures 5C, D** **)**, and we also found *EML4* exon13*-ALK* exon20 (v1) fusion in mature mRNA (**Supplementary Figure 5E**). This implies that the non-canonical *GALNT14*-*ALK* fusion was indeed *EML4* intron13*-GALNT14*-*ALK* exon19 fusion that can be spliced to form *EML4-ALK* mature mRNA (**Supplementary Figure 5F**), whereas the co-existing *SLC19A3* (IGR)-*ALK* fusion might be non-productive. Similarly, *EML4-ALK* mature mRNA were observed in patient P73, who harbored *SETD2*-*ALK* fusion at the DNA level (**Supplementary Figure 6**). Taken together, most of the newly identified non-canonical *ALK* fusions were likely to be expressed in tumors and some of them would generate the canonical *EML4-ALK* transcripts during mRNA maturation.

## Discussion

Given the promising therapeutic effects of ALK inhibitors, they are now generally used as the first-line treatment against *ALK*-positive NSCLC. As a result, identifying patients who will benefit from ALK TKIs is of great importance to improve patients’ survival and quality of life. Compared with the traditional testing methods, such as break-apart FISH or IHC, CGP could more accurately detect *ALK* fusions (21, 24–28). Besides, CGP can provide additional gene rearrangement information, such as the fusion partner and the breakage point, enabling further analyzing the correlation between the *ALK* fusion pattern and TKI therapeutic effects. In the present study, we used CGP to characterize 98 *ALK*-positive NSCLC patients and identified multiple known and novel non-canonical *ALK* fusions, most of which were likely to form functional products in tumors. In addition, we divided all 98 patients into 3 groups based on their *ALK* fusion patterns and found patients with complex *ALK* fusions had improved OS after crizotinib treatment, suggesting the *ALK* fusion pattern could be used as a prognostic marker for TKI treatment. This conclusion is supported by a recent study who found that NSCLC patients with both reciprocal and non-reciprocal *ALK* fusions had worse PFS to crizotinib treatment (29).

A few cases of co-existence of canonical and non-canonical *ALK* fusions has been reported in recent studies (29, 30); however, its clinical relevance was largely unknown. We found that there were little differences in PFS after crizotinib treatment among different *ALK* fusion groups, whereas patients with complex *ALK* fusions had better OS. This improved OS was unlikely due to confounding effects of other variables, as tested by multivariate Cox regression analysis. Intriguingly, our data showed that the complex *ALK* fusion group had trends to respond better to next-generation ALK TKIs after disease progression with crizotinib. Nevertheless, it is still unknown whether the prolonged OS in the complex *ALK* fusion group would apply to all types of ALK TKIs or whether it is due to sequentially treating patients with crizotinib and second/third-generation ALK TKIs. Recently, several next-generation ALK TKIs are being investigated as the front-line therapy rather than treating crizotinib-resistant patients (15, 16, 31). These studies generally relied on IHC and/or FISH to check *ALK* fusion status without knowing the specific fusion type. Our results suggest that it might be worth conducting these clinical trials by separating patients based on their *ALK* fusion patterns in order to figure out the optimal treatment regimen for each patient.

The mechanism of prolonged OS in patients with complex *ALK* fusions is still unknown. Although some of our preliminary data imply that different *ALK* fusion patterns may have distinct susceptibility to gain *ALK* resistant mutations after ALK TKI treatment, this result still needs to be further validated. Also, it is possible that tumors with multiple *ALK* fusions are likely to be more addicted to the ALK signaling pathway, thus making the ALK TKIs have more profound effects. Moreover, we cannot exclude the possibility that the canonical and non-canonical *ALK* fusions could be harbored by different subclones of the same tumor and these subclones could have different ALK TKI sensitivity and oncogenic potentials. By eradicating the major and more sensitive subclone using one ALK TKI, the other subclone could then thrive, which makes it a good target for subsequent treatment using another TKI. This hypothesis is supported by prolonged, although not statistically significant, PFS in complex fusion patients who treated with crizotinib and then next-generation TKIs. Therefore, the existence of *ALK* fusion subclones as well as the drug resistant mechanism should be carefully investigated using paired baseline and PD samples (32) with multi-region sequencing (33) in the future studies.

By analyzing the mutation profile, we found that some somatic genomic alterations, such as *EGFR* mutation/amplification and *CCND1* amplification, tended to be enriched in the complex *ALK* fusion group. However, these enriched mutations/CNVs were not likely to be the underlying mechanism of improved OS observed in these patients. Instead, *CCND1* amplification seems to have negative effects on post-crizotinib patient survival. Consistent with this observation, mutation/amplification of genes involved in cell-cycle control, including *CCND1*, have also been suggested to hinder the therapeutic effects of EGFR TKIs in NSCLC (34). Nevertheless, because our CGP was based on panel sequencing, whether some rare co-occurred mutations could contribute to the improved crizotinib responses still needs to be tested using whole-exome sequencing or whole genome sequencing.

There were also some limitations associated with our study: 1) The *ALK* fusion patterns were determined using 43 baseline samples and 55 post-crizotinib samples. Although the *ALK* fusion patterns were less likely to be altered by crizotinib treatment and clinical results were consistent between 43 baseline patients and all 98 patients, characterizing *ALK* fusion patterns using only baseline samples should be more accurate. 2) As this study was initiated many years ago, we used crizotinib as the major ALK TKI treatment in our cohort; however, crizotinib was no longer used as the front-line therapy in ALK-positive patients in many countries given the promising therapeutic response of next-generation TKIs. Within the 150 *ALK*-positive NSCLC patients dragonized in our hospital, 30 of them used second generation ALK inhibitors as the first TKI treatment (**Supplementary Figure 1**); however, the number of patients was limited and most of their clinical data have not matured. Therefore, we are unable to assess whether harboring complex *ALK* fusions is also a positive biomarker for front-line second-generation ALK TKIs. 3) Due to the limited availability of patient samples and the instability of RNA within the samples, we only performed RNA-seq validation for a few rare *ALK* fusions. Although the IHC positivity implies their expression in cancer cells, future studies were needed to confirm whether these rare *ALK* fusions could form functional products in the tumor. 4) The median OS in our patient cohort was significantly shorter than that in the previous studies (35). Possible reasons for this discrepancy may be due to the differences in patient ethnicity and disease stages among different studies, and our results need to be further confirmed using larger patient cohorts.

## Conclusion

Overall, we identified multiple novel non-canonical *ALK* fusions in advanced NSCLC patients, and we showed that some of the non-canonical *ALK* fusions could form canonical *EML4-ALK* transcripts during mRNA splicing. We are also the first group to comprehensively investigate the therapeutic effects of crizotinib in NSCLC patients with different *ALK* fusion patterns and demonstrated that the complex *ALK* fusions were associated with improved post-ALK TKI patient survival. Therefore, our results suggest that the determination of *ALK* fusion pattern using CGP has great clinical potentials to identify novel *ALK* fusions and make better prediction about patient prognosis.

## Data Availability Statement

The datasets presented in this study can be found in online repositories. The names of the repository/repositories and accession number(s) can be found below: NODE (http://www.biosino.org/node), accessions OEP001261 and OEP001269.

## Ethics Statement

The studies involving human participants were reviewed and approved by the institutional ethics review board of Guangdong Provincial People’s Hospital. The patients/participants provided their written informed consent to participate in this study.

## Author Contributions

Conception and design were done by J-JY, JK, and Y-LW. J-JY, JK, H-JC, and X-CZ provided the study materials or patients. W-ZZ, JS, QZ, H-YT, ZW, C-RX, X-NY, and Z-HC collected and assembled the data. Data analysis was done by JK, YX, XW, XZ, and YS. The manuscript was written by JK and J-JY. All authors contributed to the article and approved the submitted version.

## Funding

This work was supported by the High-level Hospital Construction Project (Grant No. DFJH201809, J-JY), National Natural Science Foundation of China (Grant No. 81972164, J-JY), Natural Science Foundation of Guangdong Province (Grant No. 2019A1515010931, J-JY), National Key Technology R&D Program of the Ministry of Science and Technology of China: Prevention and Control of Major Non-communicable Diseases (Grant No. 2016YFC1303304, J-JY), Key Lab System Project of Guangdong Science and Technology Department-Guangdong Provincial Key Lab of Translational Medicine in Lung Cancer (Grant No. 2017B030314120, Y-LW), and Strategic Priority Research Program of the Chinese Academy of Sciences (Grant No. XDA12020103 to X-CZ and JA, and grant No. XDA12020105 to X-CZ and A-JS). 

## Conflict of Interest

XZ and YS are the employees of Nanjing Geneseeq Technology Inc.; YX and XW are the employees of Geneseeq Technology Inc.

The remaining authors declare that the research was conducted in the absence of any commercial or financial relationships that could be construed as a potential conflict of interest.

## Supplementary Material

Supplementary Figure 1 An overview of the study design. The flowchart for including advanced NSCLC patients for the retrospective study.

Supplementary Figure 2 The pie diagram illustrating the percentage of patients in each *ALK* fusion group for 43 patients with baseline CGP **(A)** or for all 98 *ALK*-positive patients with either baseline or progressive disease CGP **(B)**.

Supplementary Figure 3 Paired baseline (before crizotinib treatment) and PD (disease progression after crizotinib treatment) mutations/CNVs of NSCLC patients with different *ALK* fusion patterns. N = 17. BL, baseline.

Supplementary Figure 4 Pre-mature mRNA detected by RNA-seq revealed complex *ALK* fusions. **(A)** *ALK* intron1-*ALK* intron19 fusion was detected in pre-mature mRNA by RNA-seq; **(B)** *EML4* intron6-*ALK* intron1 fusion was detected in pre-mature mRNA by RNA-seq.

Supplementary Figure 5
*GALNT14*-*ALK* and *SLC19A3* (IGR)-*ALK* fusions detected by CGP in one NSCLC patient (P62) resulted in a classic *EML4-ALK* fusion mRNA only. **(A)** *GALNT14*-*ALK* fusion was detected at the DNA level by DNA-seq. **(B)** *SLC19A3* (IGR)-*ALK* fusion was detected at the DNA level by DNA-seq. **(C)** *EML4-GALNT14* fusion was detected in pre-mature mRNA by RNA-seq. **(D)** *GALNT14*-*ALK* fusion was detected in pre-mature mRNA by RNA-seq. **(E)** Mature *EML4-ALK* v1 fusion was detected at the RNA level by mRNA-Seq. **(F)** Model for stepwise *EML4-ALK* fusion formation during gene transcription.

Supplementary Figure 6
*SETD2*-*ALK* fusion detected by CGP in an NSCLC patient (P73) resulted in a classic *EML4-ALK* fusion mRNA. **(A)** *EML4-SETD2* fusion was detected in pre-mature mRNA by RNA-seq. **(B)** *SETD2*-*ALK* fusion was detected in pre-mature mRNA by RNA-seq. **(C)** Mature *EML4-ALK* v5a fusion was detected at RNA levels by mRNA-seq. **(D)** Model for stepwise *EML4-ALK* fusion formation during gene transcription.
