# Supplementary figures and images for: Complex ALK Fusions Are Associated With Better Prognosis in Advanced Non-Small Cell Lung Cancer
Source: Front Oncol. 2020 Dec 11;10:596937. doi: 10.3389/fonc.2020.596937 (PMC7759679; doi:10.3389/fonc.2020.596937)

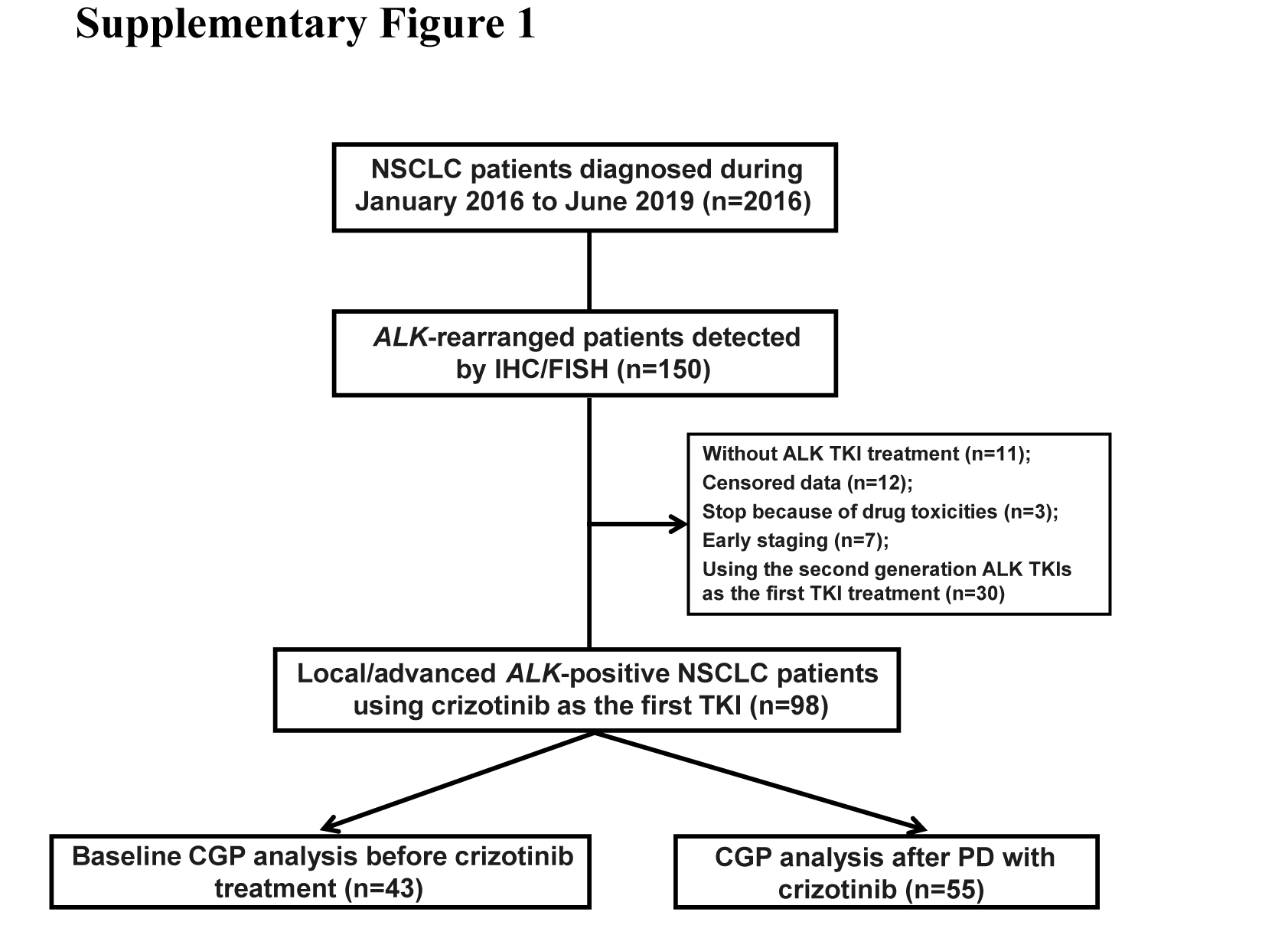

Supplement: Supplementary Figure 1 — An overview of the study design. The flowchart for including advanced NSCLC patients for the retrospective study. [file Image_1.tif]

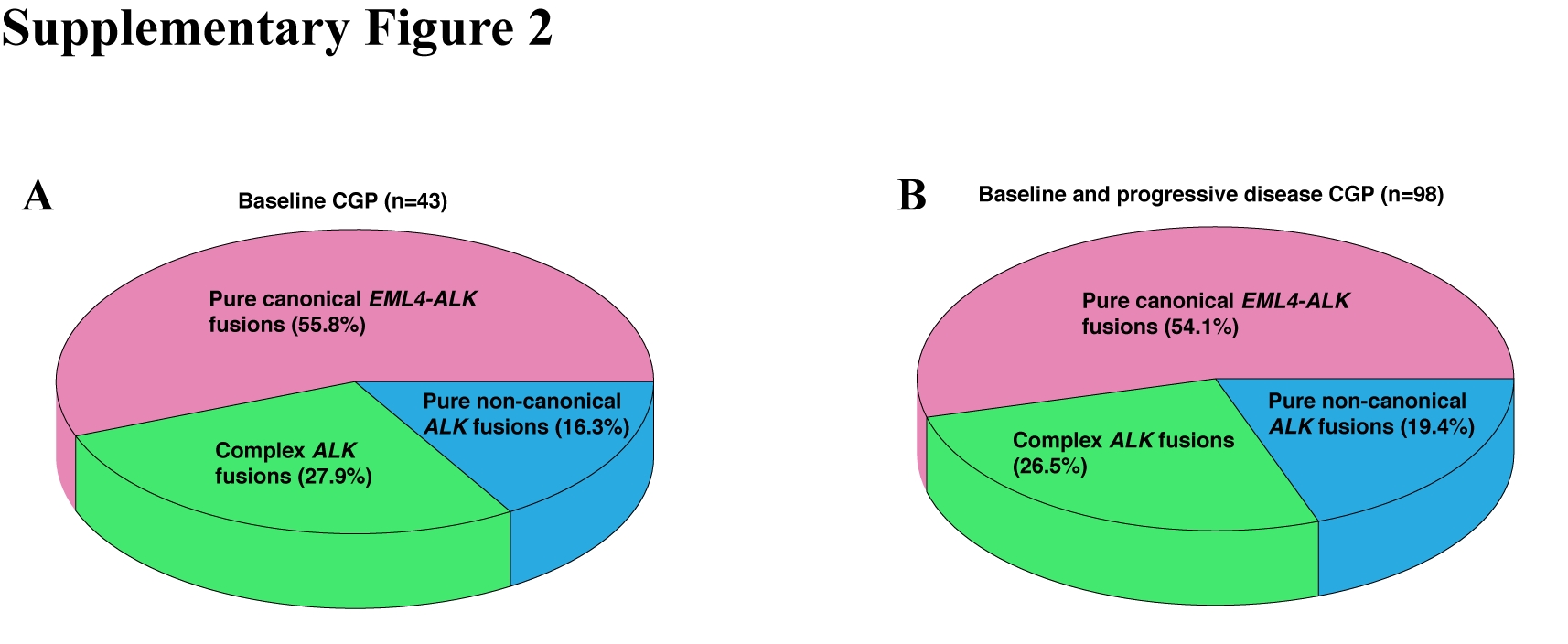

Supplement: Supplementary Figure 2 — The pie diagram illustrating the percentage of patients in each ALK fusion group for 43 patients with baseline CGP (A) or for all 98 ALK-positive patients with either baseline or progressive disease CGP (B). [file Image_2.tif]

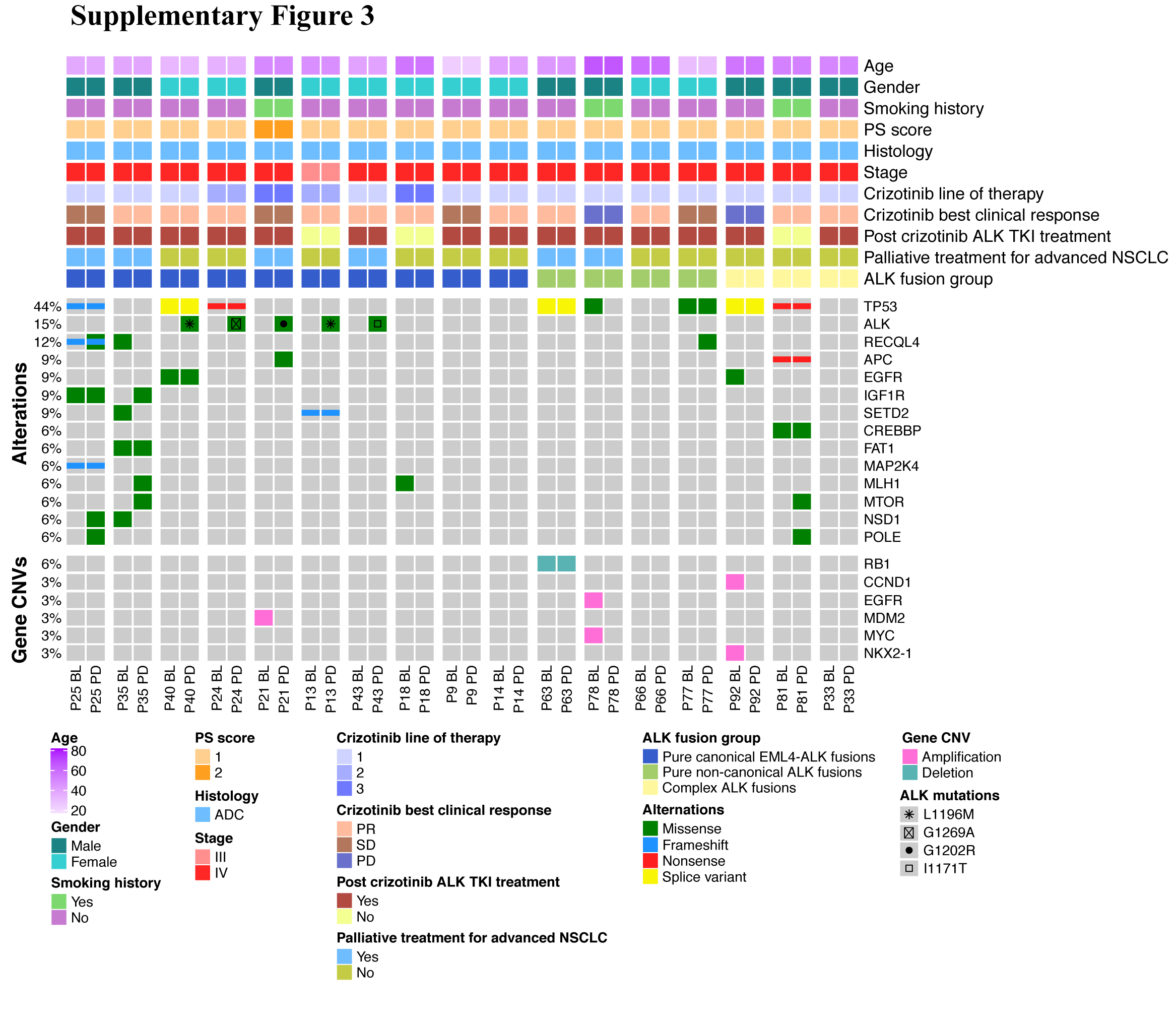

Supplement: Supplementary Figure 3 — Paired baseline (before crizotinib treatment) and PD (disease progression after crizotinib treatment) mutations/CNVs of NSCLC patients with different ALK fusion patterns. N = 17. BL, baseline. [file Image_3.tif]

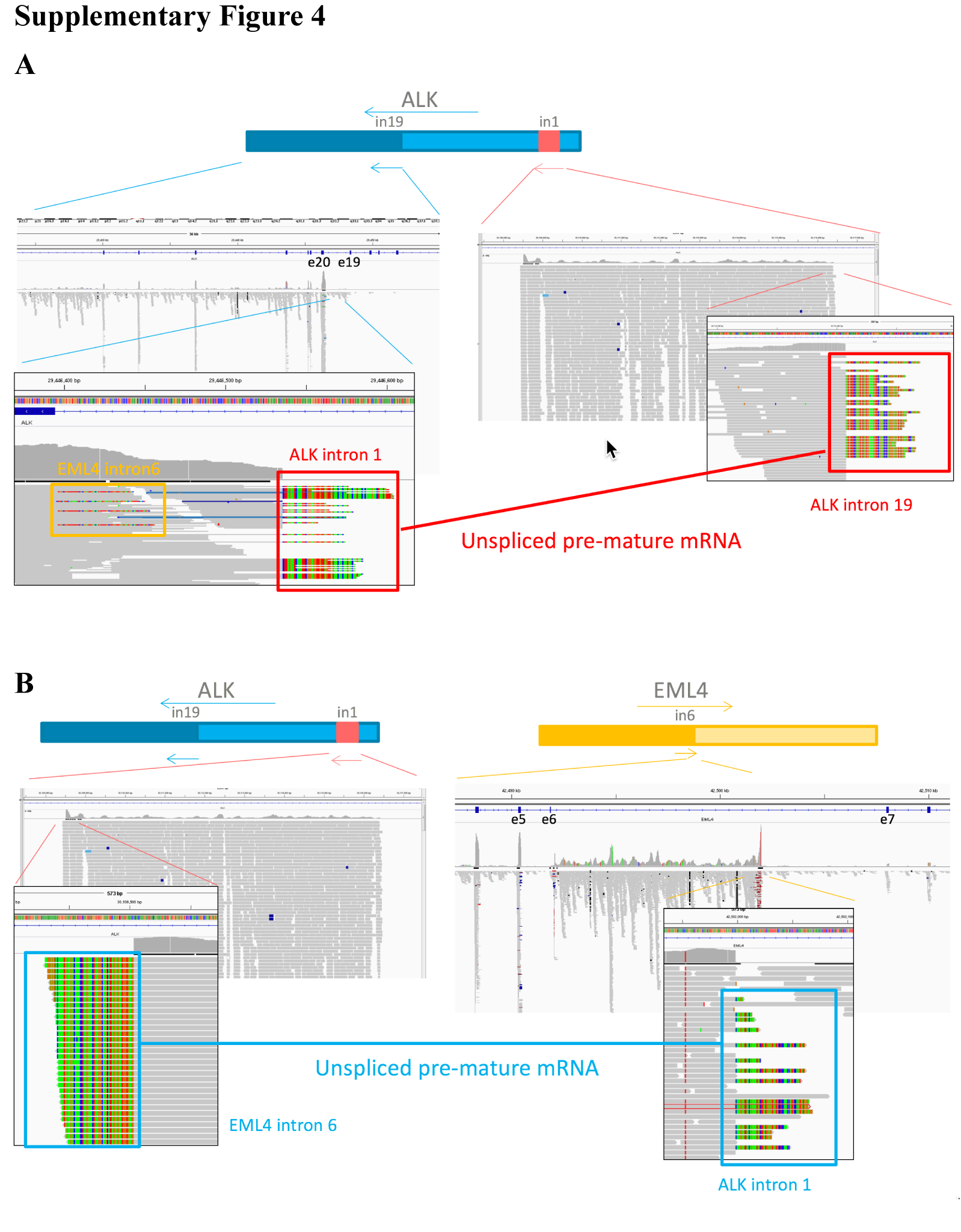

Supplement: Supplementary Figure 4 — Pre-mature mRNA detected by RNA-seq revealed complex ALK fusions. (A) ALK intron1-ALK intron19 fusion was detected in pre-mature mRNA by RNA-seq; (B) EML4 intron6-ALK intron1 fusion was detected in pre-mature mRNA by RNA-seq. [file Image_4.tif]

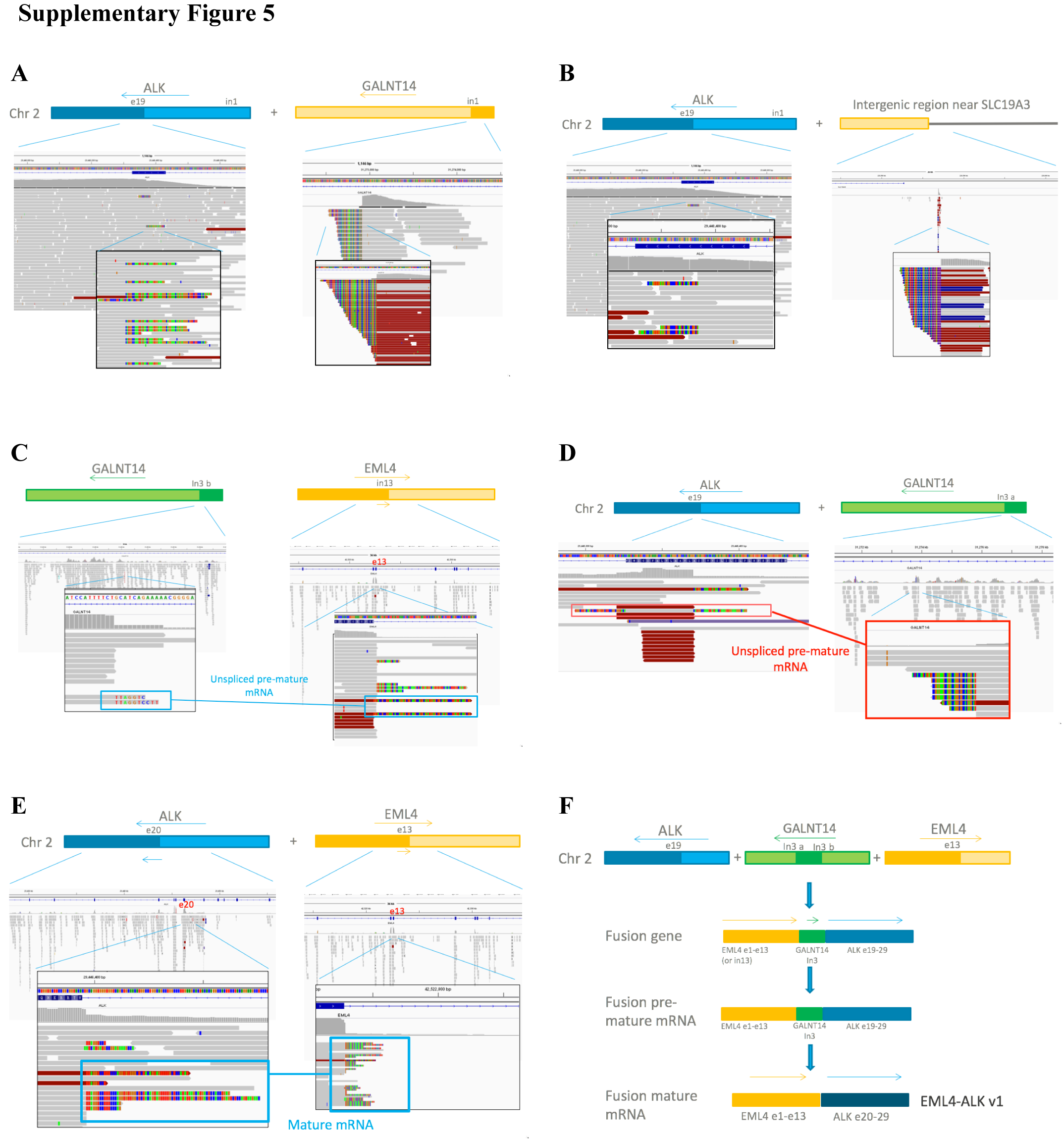

Supplement: Supplementary Figure 5 — GALNT14-ALK and SLC19A3 (IGR)-ALK fusions detected by CGP in one NSCLC patient (P62) resulted in a classic EML4-ALK fusion mRNA only. (A) GALNT14-ALK fusion was detected at the DNA level by DNA-seq. (B) SLC19A3 (IGR)-ALK fusion was detected at the DNA level by DNA-seq. (C) EML4-GALNT14 fusion was detected in pre-mature mRNA by RNA-seq. (D) GALNT14-ALK fusion was detected in pre-mature mRNA by RNA-seq. (E) Mature EML4-ALK v1 fusion was detected at the RNA level by mRNA-Seq. (F) Model for stepwise EML4-ALK fusion formation during gene transcription. [file Image_5.tif]

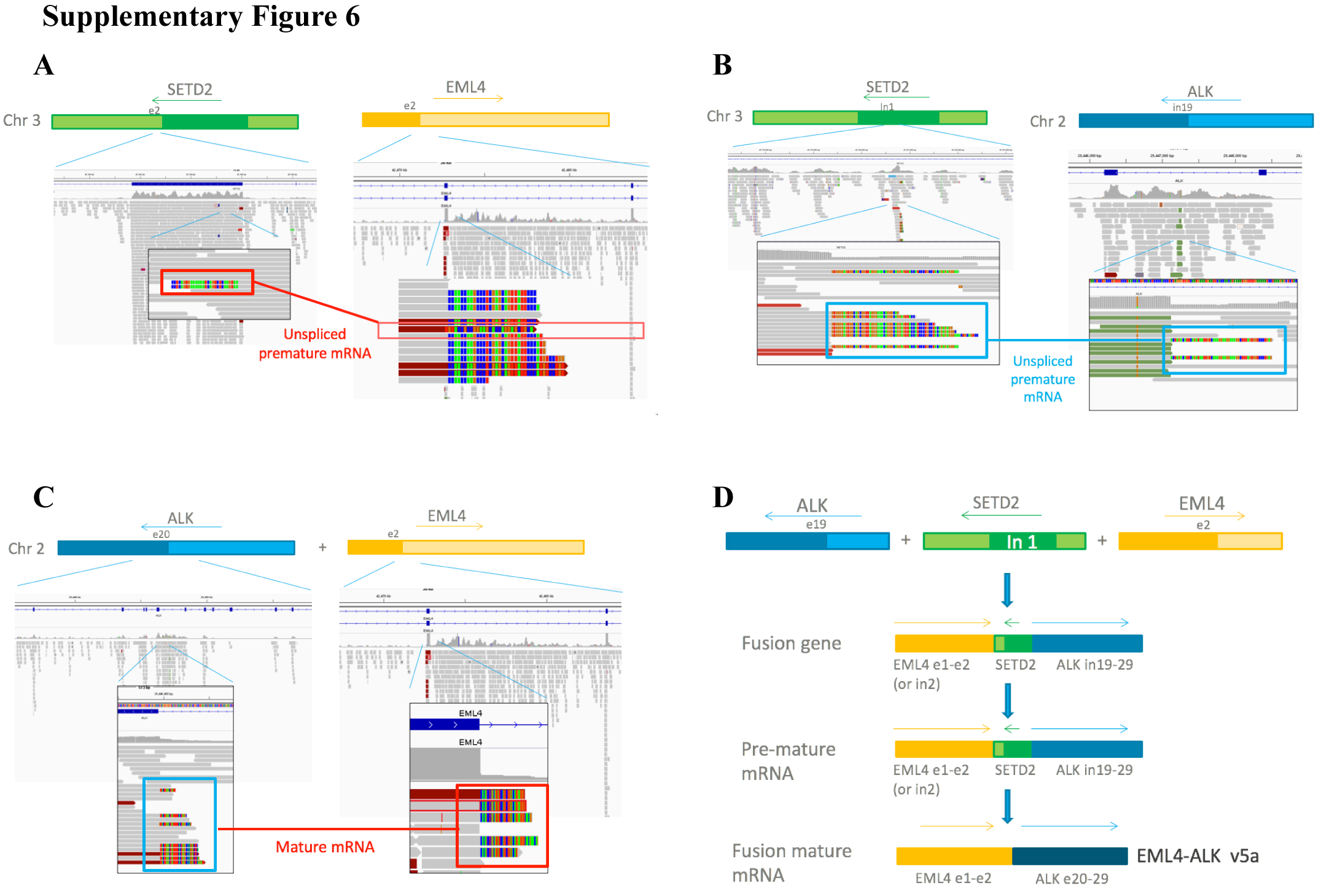

Supplement: Supplementary Figure 6 — SETD2-ALK fusion detected by CGP in an NSCLC patient (P73) resulted in a classic EML4-ALK fusion mRNA. (A) EML4-SETD2 fusion was detected in pre-mature mRNA by RNA-seq. (B) SETD2-ALK fusion was detected in pre-mature mRNA by RNA-seq. (C) Mature EML4-ALK v5a fusion was detected at RNA levels by mRNA-seq. (D) Model for stepwise EML4-ALK fusion formation during gene transcription. [file Image_6.tif]
